# Running Reduces Uncontrollable Stress-Evoked Serotonin and Potentiates Stress-Evoked Dopamine Concentrations in the Rat Dorsal Striatum

**DOI:** 10.1371/journal.pone.0141898

**Published:** 2015-11-10

**Authors:** Peter J. Clark, Jose Amat, Sara O. McConnell, Parsa R. Ghasem, Benjamin N. Greenwood, Steven F. Maier, Monika Fleshner

**Affiliations:** 1 Integrative Physiology, University of Colorado Boulder, 354 UCB, Boulder, CO, 80309, United States of America; 2 Department of Psychology & Neuroscience, University of Colorado Boulder, Muenzinger D244, 345 UCB, Boulder, CO, 80309, United States of America; 3 Department of Psychology, University of Colorado Denver, Campus Box 173, PO 173364, Denver, CO, 80217–3364, United States of America; Technion - Israel Institute of Technology, ISRAEL

## Abstract

Accumulating evidence from both the human and animal literature indicates that exercise reduces the negative consequences of stress. The neurobiological etiology for this stress protection, however, is not completely understood. Our lab reported that voluntary wheel running protects rats from expressing depression-like instrumental learning deficits on the shuttle box escape task after exposure to unpredictable and inescapable tail shocks (uncontrollable stress). Impaired escape behavior is a result of stress-sensitized serotonin (5-HT) neuron activity in the dorsal raphe (DRN) and subsequent excessive release of 5-HT into the dorsal striatum following exposure to a comparatively mild stressor. However, the possible mechanisms by which exercise prevents stress-induced escape deficits are not well characterized. The purpose of this experiment was to test the hypothesis that exercise blunts the stress-evoked release of 5-HT in the dorsal striatum. Changes to dopamine (DA) levels were also examined, since striatal DA signaling is critical for instrumental learning and can be influenced by changes to 5-HT activity. Adult male F344 rats, housed with or without running wheels for 6 weeks, were either exposed to tail shock or remained undisturbed in laboratory cages. Twenty-four hours later, microdialysis was performed in the medial (DMS) and lateral (DLS) dorsal striatum to collect extracellular 5-HT and DA before, during, and following 2 mild foot shocks. We report wheel running prevents foot shock-induced elevation of extracellular 5-HT and potentiates DA concentrations in both the DMS and DLS approximately 24 h following exposure to uncontrollable stress. These data may provide a possible mechanism by which exercise prevents depression-like instrumental learning deficits following exposure to acute stress.

## Introduction

The stress response is an adaptive physiological reaction to challenges that functions to promote survival. Protracted or exaggerated responses to chronic or severe stress, however, can become maladaptive to brain function, playing a well-documented role in the development of stress-related disorders like anxiety and depression. Mounting evidence indicates abnormal levels of monoamine neurotransmitters contribute to the expression of symptoms associated with anxiety and depression, however the neurobiological mechanisms involved are not well understood (for review see [1–4]). On the other hand, regular mild to moderate physical activity can be beneficial for cognitive function and mental health [5, 6]. For instance, it is clear that physically active organisms are stress robust. Indeed, accumulating evidence in human and rodent studies indicates that a physically active lifestyle promotes stress-resistance and resilience, or respectively, the capability to endure and rapidly recover from the damaging consequences of stress [5, 7–13]. While the specific mechanisms are unclear, exercise may buffer stress, in part, by altering the signaling of brain monoamines [14]. Indeed, acute bouts of exercise have been shown to enhance the activity of monoamines including serotonin (5-HT) and dopamine (DA) in a number of stress sensitive rodent brain regions [14–18]. Moreover, data from rodent models suggest engaging in long-term exercise induces plasticity in 5-HT and DA systems within stress responsive areas that may influence the activity of these neurotransmitters in ways that promote stress-resistance [10, 19–22]. A better understanding of the mechanisms by which physical activity promotes stress robustness could lead to the discovery of novel targets to treat or prevent stress related disorders.

Insight into the neural mechanisms by which physical activity promotes stress resistance has come from rodent models where physical activity status is manipulated by providing access to running wheels before exposure to a potent stressor; 100 inescapable and unpredictable tail shocks (acute stress) [5, 7, 8]. Animals with access to running wheels are protected from the development of several anxiety- and depression-like behaviors following exposure to this acute stressor, including exaggerated fear, increased drug seeking behavior, reduced social exploration, and shuttle box learning deficits [10, 23, 24]. In particular, shuttle box escape deficits following acute stress are thought to be a consequence of impaired goal directed instrumental behavior, a common symptom of depression [25, 26]. The circuitry underlying impaired acquisition of shuttle box task has been well characterized. Exposure to tail shocks hyper-activates 5-HT neurons in the dorsal raphe nucleus (DRN) [27, 28] desensitizing the inhibitory 5-HT_1A_ autoreceptors (5-HT_1A_R) [29, 30], thereby removing an important source of inhibition over the activity of these neurons. 5-HT activity, thus, becomes sensitized in areas innervated by the DRN during subsequent exposure to a comparatively mild stressor [25, 28, 31, 32]. Impaired escape behavior following acute stress is related to a potent rise in extracellular 5-HT concentrations in DRN projection site the dorsal striatum, and can be prevented by pharmacological blockade of 5-HT_2C_ receptors (5-HT_2C_R) located in the same striatal region [25]. These data specifically highlight the significance of a stress-sensitized DRN 5-HT response acting in the dorsal striatum to produce depression-like escape deficits. While much is known about the neurobiology underlying the development of escape deficits following exposure to this stressor, comparatively less is understood about how exercise prevents such impairments.

Wheel running may prevent the development of acute stress-induced escape deficits by constraining the activity of DRN 5-HT neurons. Indeed, evidence from our lab suggests rats that engage in 6 wk (but not 3 wk) voluntary running display reduced expression of neural activity marker c-Fos in DRN 5-HT neurons during exposure to tail shocks [10, 19]. Reduced 5-HT neuron activity in wheel running rats is paralleled by an up regulation in 5-HT_1A_R in the DRN [10, 19]. A potentially stronger 5-HT_1A_R-mediated inhibition of DRN 5-HT neurons in running rats during acute stress may be sufficient to attenuate or eliminate the subsequent stress-induced sensitization of 5-HT activity in the dorsal striatum, thereby conferring protection against depression-like escape deficits [7]. However, it is currently unknown if exercise prevents or attenuates stress-sensitized 5HT activity in the dorsal striatum following exposure to acute stress.

The purpose of this experiment was to test the hypothesis that 6 weeks of running prevents stress-sensitized 5-HT levels in the rat dorsal striatum using *in vivo* microdialysis, given the previously reported increases of 5-HT_1A_R expression and reduced neural activity in the DRN [10, 19]. The dorsal striatum was selectively targeted because previous work from our lab suggests acute stress-sensitized 5-HT response in this brain region is necessary for shuttle box escape deficits [25], while the contributions of other striatal sub-structures to this paradigm remains unknown. In addition, DA signaling in the striatum is critical for goal-directed processes including instrumental learning and can be modulated by excessive 5-HT activity [33–37]. Therefore, we also explored the possibility that exercise alters DA release in the dorsal striatum following stress exposure in a manner consistent with protection against impaired shuttle box behavior. Sampling was performed in both the medial (DMS) and lateral (DLS) dorsal striatum, as evidence suggests these regions each modulate a distinct aspect of the instrumental behavior, such as shuttle box escape. Indeed, DMS is necessary for the initial acquisition of instrumental behaviors, whereas the DLS then becomes selectively recruited to maintain an instrumental process once it is acquired [38–46]. Therefore, stress induced changes to 5-HT or DA concentrations that are consistent with development of, or protection against, impaired escape behavior may be present in only one sub-region such as the DMS, since rats fail in the acquisition stage of the shuttle box task following acute stress exposure. Moreover, contrasting responses to unpredictable stress have been reported in sub regions of the rat dorsal striatum, whereby DMS displayed atrophy and the DLS displayed hypertrophy [47], suggesting these areas may react differently to stressors. We report that long-term wheel running attenuates acute stress-induced sensitization of 5-HT, but potentiates DA concentrations in both the DMS and DLS following subsequent exposure to foot shock. These data may provide insight into possible neural adaptations from exercise that contribute to the prevention of depression-like instrumental learning deficits following stress.

## Materials and Methods

### Subjects and husbandry

Upon arrival, 70 male Fischer344 rats (obtained from Harlan SPF, Indianapolis, IN, USA) weighing approximately 148g were individually housed in standard laboratory cages (45×25.2×14.7 cm) or cages with locked running wheels (45×25.2×14.7 cm). After 1 week, running wheels were unlocked and rats received free access to wheels for the remainder of the study (6–7 weeks). The time course of wheel access was chosen because 6, but not 3, weeks of running is sufficient to prevent shuttle box escape deficits following exposure to 100 unpredictable and inescapable tail shocks [10, 48]. Daily wheel revolutions were recorded digitally using Vital View software (Mini Mitter, Bend, OR, USA). A total of 12 rats were removed from analysis during the experiment due to surgical complications, improper cannula placement, or damage to dialysis probe during sampling. Group sample sizes used for statistical analysis are reported below. Rooms were controlled for temperature (21 ± 1°C) and photo-period (12:12 L:D) for the entire study. Food (Harlan Teklad 7012) and water were provided *ad libitum*. All procedures were approved by the University of Colorado Institutional Animal Care and Use Committee and adhered to NIH guidelines. Special care was taken to minimize animal discomfort during all procedures.

### Cannulae Implantation

During the 3^rd^ or 4^th^ week of voluntary wheel running or sedentary conditions rats underwent stereotaxic surgery for microdialysis guide cannulae implantation. Cannulae implantation was performed during the 3^rd^ and 4^th^ week of running because previous work from our lab indicates exercising rats that undergo surgery during this period are still protected from behavioral deficits dependent that follow unpredictable and inescapable tail shock [25]. Rats were anesthetized with a cocktail of ketamine (0.75 mg/kg i.p.; Vedco, St. Joseph, MO, USA) and medetomidine (0.5 mg/kg i.p.; Pfizer, New York, NY, USA). Guide cannulae were implanted unilaterally in the right or left DMS (-0.2 A/P, ± 2 M/L, -3.8 D/V from bregma) or DLS (-0.2 A/P, ± 4.3 M/L, -4.5 D/V from bregma), based on the Paxinos and Watson atlas. Rats were immunized with a single subcutaneous injection of penicillin (0.25mL/Kg; of Combi-Pen, Agrilbas, St. Joseph, Missouri, USA). Atipamezole (0.5 mg/kg i.p.; Pfizer, New York, NY, USA) was administered following surgery to reverse the effects of medetomidine.

#### Tail shock procedure (acute stress)

Approximately 3–4 weeks after surgery, rats were randomly assigned to either receive tail shock stress (DMS: n = 8 Sedentary Stress, n = 8 Running Stress; DLS: n = 7 Sedentary Stress, n = 8 Running Stress) or no stress (DMS: n = 7 Sedentary Control, n = 7 Running Control; DLS: n = 7 Sedentary Control, n = 6 Running Control). Rats that received tail shock were restrained in a Plexiglas box with the tail protruding from the back of the box where electrodes were placed to deliver 100 tail shocks (5 s, 1.5 mA, 1 min ISI). This uncontrollable stress procedure is necessary for hyperactivation of the DRN, and a sensitized 5-HT response following subsequent mild stress exposure. Control rats were left undisturbed in home cages during tail shocks.

### Microdialysis

Approximately 4 h following tail shocks, microdialysis probes (CMA 12, 2mm) were inserted into guide cannulae and rats were placed in separate Plexiglas dialysis bowls (Bioanalytical Systems) with access to food and water overnight. Artificial cerebral spinal fluid was perfused through probes using an infusion pump at a follow rate of 0.2 ul/min overnight. The next morning flow rate was increased to 1.5 ul/min and after 90 min of equilibration sampling began. Infusion rate remained constant throughout the sample collecting. Samples were collected every 20 mins. The first two samples (B1-B2) were collected in dialysis bowls. Rats were then carefully moved from dialysis bowls to conditioning chambers where remaining samples were collected before (B3-B4), and immediately following (FS-P4) 2 foot shocks (5s, 0.8 mA, 1min ISI).

### HPLC

Dialysates containing extracellular 5-HT and DA were measured by high-performance liquid chromatography (HPLC) with electrochemical detection. The system consisted of an ESA 5600A Coularray detector with an ESA 5014B analytical cell and an ESA 5020 guard cell. The column was an ESA MD-150 (C-18, 3 m, 150 3.2 mm) maintained at 38°C, and the mobile phase was the ESA buffer MD-TM. The analytical cell potentials were kept at -100 mV and +200 mV and the guard cell at +220mV. Dialysate (25 μl) was injected with an ESA 542 autosampler that kept the dialysates at 6°C. External standards (Sigma) were run for each daily analysis to quantify 5-HT and DA. Samples that contained low volumes of dialysate or were lost during collection were not included for analysis.

### Probe placement

**Fig 1:**
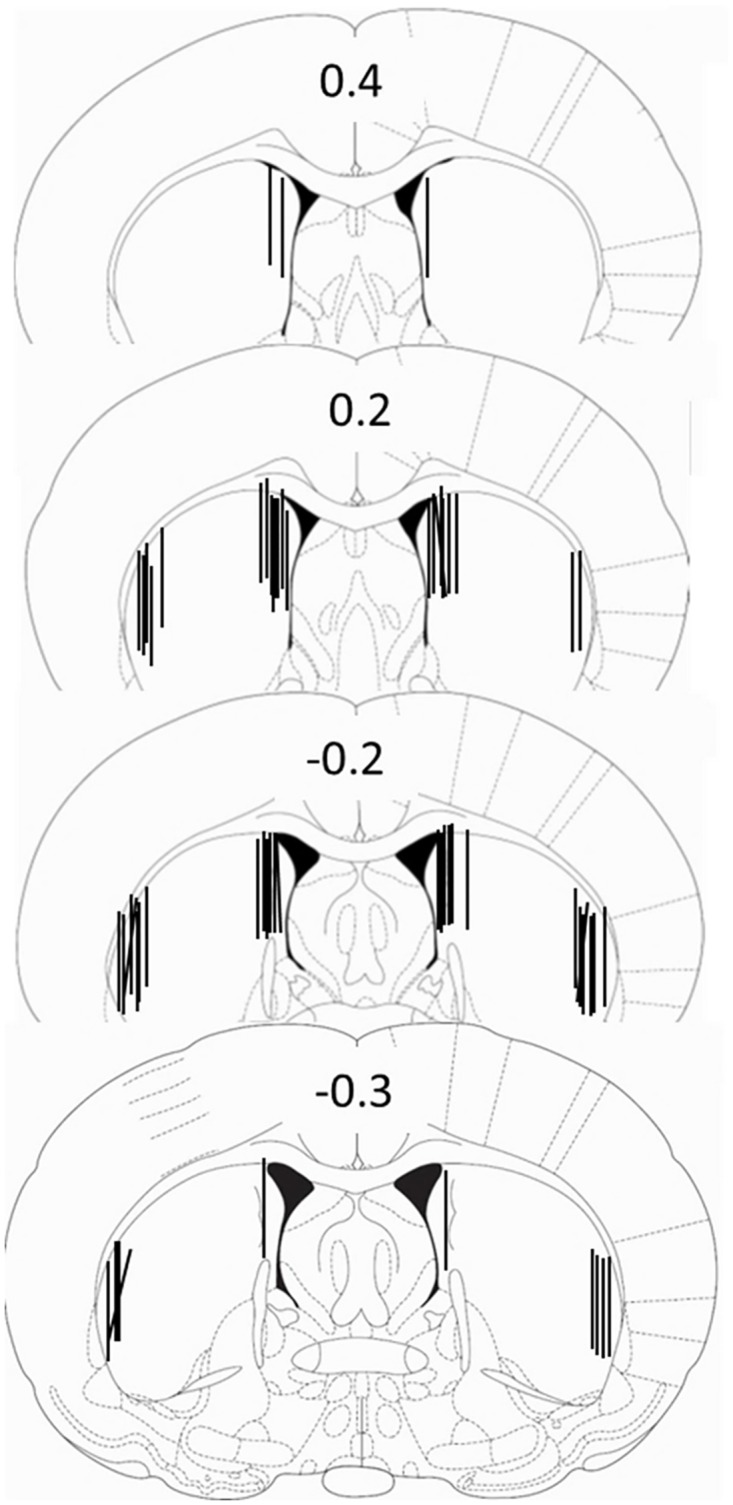
Cannula placement. Reconstruction of microdialysis probe placement in the medial and lateral dorsal striatum of rats used for analysis. Illustrations adapted from Paxinos and Watson.

Following completion of microdialysis rats were euthanized by rapid decapitation. Brains were rapidly extracted and froze in isopentane (-20C). Brains were coronal sectioned at 40 μm and Nissl stained to verify cannulae placement. Misplaced cannulae were excluded from analysis. Fig 1 displays approximate locations of dialysis probes for the rats included in data analysis.

### Data analysis

Overall distance run was compared between Running Stress and Running Control rats using Student’s t-test. Average baseline (B1-B4) values of 5HT and DA in the DMS and DLS were compared by 2-way ANOVAs with exercise condition (Sedentary vs. Running) and acute stress treatment (Control vs. Stress) as factors. Samples containing outlier values were removed from analysis, as determined by Grubbs test. The remaining data are presented as a percentage of baseline mean, as completed by dividing a sampling period (B1-P4) by the mean of the 4 baseline samples (B1-B4) for each rat (as previously reported in [25, 31, 32]). Data were analyzed using a 3-way repeated measure ANOVA with exercise condition, stress treatment, and individual sampling periods (B1-P4) as factors. In addition, average concentrations of 5HT and DA over the entire sampling period following foot shock (FS-P4) were compared using a 2-way ANOVA with exercise condition and stress treatment as factors. One group *t-tests* were performed to determine if the mean percentage change in extracellular 5HT or DA concentrations following foot shock (FS-P4) differed from baseline. *Post hoc* analyses with Tukey-Kramer corrections were completed when appropriate. In all analyses, *P* < 0.05 was considered statistically significant.

## Results

### Wheel running

Average distance traveled on wheels for the entire time course of this experiment was 3.33 km/day (± 0.24 SE). During the week of surgery (weeks 3 or 4), running distance fell to an average of 1.36 km/day (± 0.36 SE). However, daily running distance recovered during weeks 5 and 6 to 3.99 (± 0.64 SE) and 3.77 km/day (± 0.57 SE) respectively. No differences in running distance were observed between Control and Stress groups.

### Baseline values of 5HT & DA

Table 1 shows the average basal (B1—B4) values of 5-HT and DA. No significant differences were found between groups for 5HT or DA concentrations in the DMS and DLS.

**Table 1:** Values of average baseline (B1-B4) samples for each group. Values are group means (SEM) expressed in pg/ul.

	Sedentary	Sedentary	Running	Running
	Control	IS	Control	IS
DMS 5HT	0.23 (0.04)	0.24 (0.05)	0.18 (0.04)	0.26 (0.1)
DA	1.3 (0.3)	1.7 (0.8)	2.7 (0.6)	1.7 (0.5)
DLS 5HT	0.22 (0.07)	0.28 (0.07)	0.22 (0.07)	0.21 (0.07)
DA	1.8 (0.4)	3.6 (0.8)	3.1 (0.6)	2.2 (0.7)

### Serotonin

#### Dorsal medial striatum

**Fig 2:**
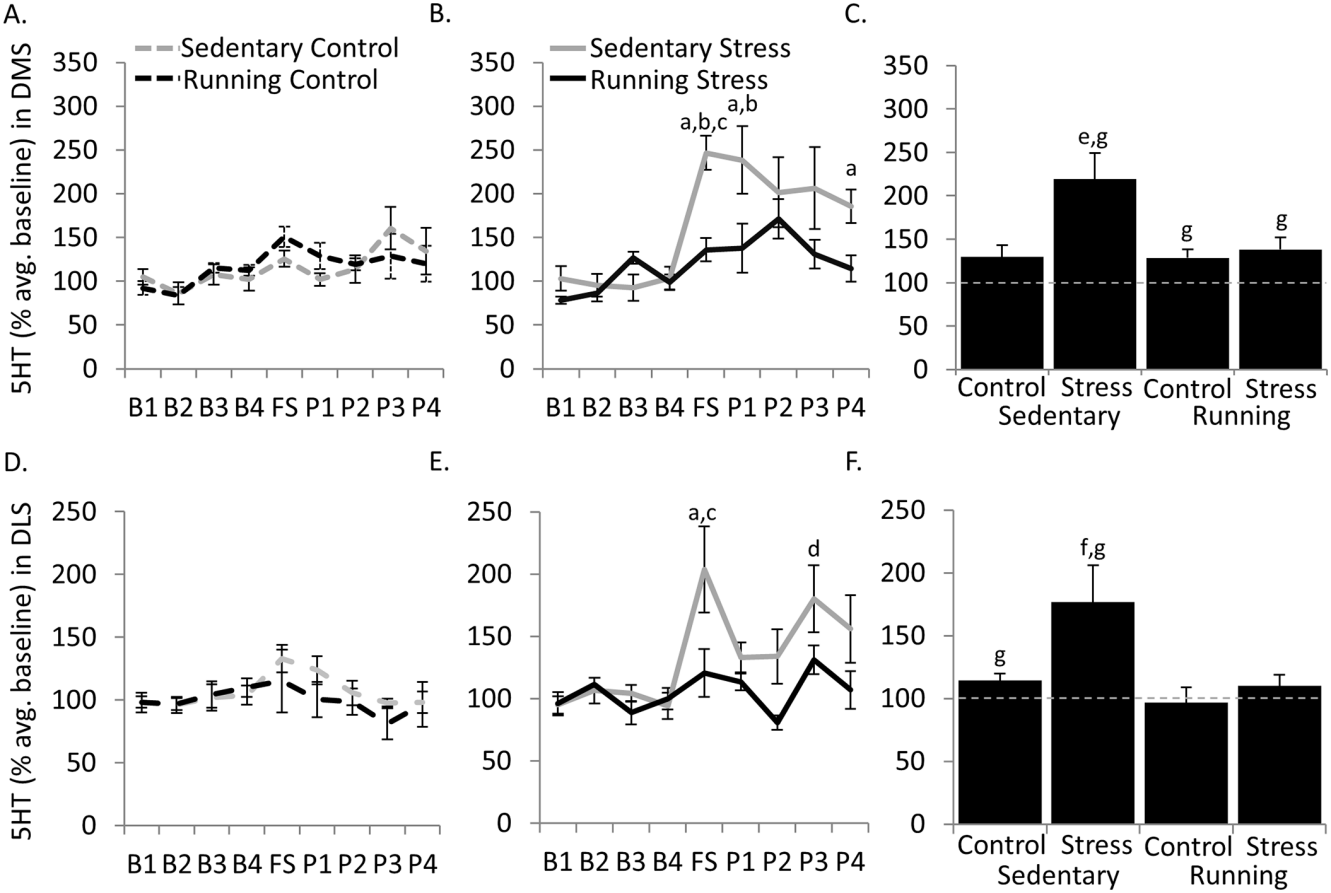
Serotonin concentrations in the dorsal striatum. A) Mean extracellular 5-HT levels expressed as a percentage of baseline samples in the dorsal medial striatum (DMS) of control rats during each 20 min sampling period. B) same as A but for rats exposed to uncontrollable stress (Stress). C) Combined mean post-shock (FS-P4) extracellular 5-HT levels expressed as a percentage of baseline samples (*grey dashed line*) in the DMS for each group. D) Mean extracellular 5-HT levels expressed as a percentage of baseline samples in the dorsal lateral striatum (DLS) of control rats during each 20 min sampling period. E) same as D but for rats exposed to uncontrollable stress (Stress). F) Combined mean post-shock (FS-P4) extracellular 5-HT levels expressed as a percentage of baseline samples (*grey dashed line*) in the DLS for each group. ^a^ *P*<0.01 different from B1, B2, B3, or B4 in Sedentary Stress; ^b^ *P*<0.05 Sedentary Stress from Sedentary Control at FS and P1 sampling periods; ^c^ *P*<0.05 Sedentary Stress from Running Stress at FS sampling period; ^d^ *P*<0.05 P3 from B1 or B4 in Sedentary Stress group; ^e^ *P*<0.05 Sedentary Stress from Sedentary Control, Running Control, and Running Stress; ^f^ *P*<0.05 from Running Control and Running Stress; ^g^ *P*<0.05 from baseline (*grey dashed line*)

For individual sampling periods, a significant 3-way interaction between exercise condition, stress, and sample period was found [F(25, 213) = 2.14, *P* = 0.002] (Fig 2A and 2B). Within groups, post hoc analysis revealed Sedentary Stress rats had greater concentrations of 5HT during FS, P1, and P4 than all baseline (B1, B2, B3, or B4) sampling periods (all *P*<0.01). No other within group sampling points differed. Post hoc analysis also revealed no differences between groups during B1-B4 sampling periods. However, Sedentary Stress maintained greater concentrations of 5HT compared to Sedentary Control at FS (*P* = 0.03) and P1 (*P* = 0.005) sampling periods. Exercise prevented the sensitized 5HT efflux following foot shock in rats that received tail shock, as Running Stress maintained lower concentrations of 5-HT than Sedentary Stress rats during the FS sample period (*P* = 0.01).

Averaging across the post-foot shock sampling periods, a significant 2-way interaction was found between exercise and stress conditions [F(1,26) = 4.23, *P*<0.05] (Fig 2C). Post hoc analysis revealed that Sedentary Stress group had greater post-shock concentrations of 5-HT than Sedentary Control (*P* = 0.03), Running Control (*P* = 0.03), and Running Stress (*P*<0.05) groups. One group *t-tests* revealed that extracellular 5-HT concentrations tended to be greater during post-foot shock periods than baseline for Sedentary Stress [*t*(7) = 3.92, *P* = 0.006], Running Control [*t*(6) = 2.94, *P* = 0.03], and Running Stress [*t*(7) = 2.71, *P* = 0.03] groups, however a non-significant trend was also observed for Sedentary Control (*P* = 0.08) (Fig 2C).

#### Dorsal lateral striatum

For individual sampling periods, a significant 3-way interaction between exercise condition, stress, and sample period was found (Fig 2E and 2F) [F(25, 192) = 2.00, *P* = 0.005]. Within groups, post hoc analysis revealed Sedentary Stress rats had greater concentrations of 5HT during FS than all baseline (B1, B2, B3, or B4) sampling periods (All *P*<0.002). Moreover, Sedentary Stress rats had greater concentrations of 5HT during P3 than B1 (*P*<0.05) and B4 (*P* = 0.03) sampling periods. No other within group sampling points differed significantly. Post hoc analysis revealed no differences between groups during B1-B4 sampling periods. However, exercise prevented the elevated 5-HT efflux following foot shock in rats that received tail shock, as Running Stress maintained lower concentrations of 5-HT than Sedentary Stress rats during the FS sample period (*P*<0.05).

Averaging across the post-foot shock sampling periods, significant main effects of exercise [F(1, 24) = 6.68, *P* = 0.02] and stress [F(1, 24) = 5.38, *P* = 0.03], but no significant interaction between exercise and stress (*P* = 0.1) was found (Fig 2F). Post hoc analysis revealed that Sedentary Stress group maintained greater post-shock concentrations of 5-HT than Running Control (*P* = 0.02), Running Stress (*P*<0.05), and a non-significant trend towards increased levels compared to Sedentary Control (*P* = 0.07) groups. One group *t-tests* revealed that extracellular 5-HT concentrations tended to be greater during post-foot shock periods than baseline in Sedentary Control [*t*(7) = 2.68, *P* = 0.04] and Sedentary Stress [*t*(7) = 2.64,*P* = 0.04] groups (Fig 2F).

### Dopamine

#### Dorsal medial striatum

**Fig 3:**
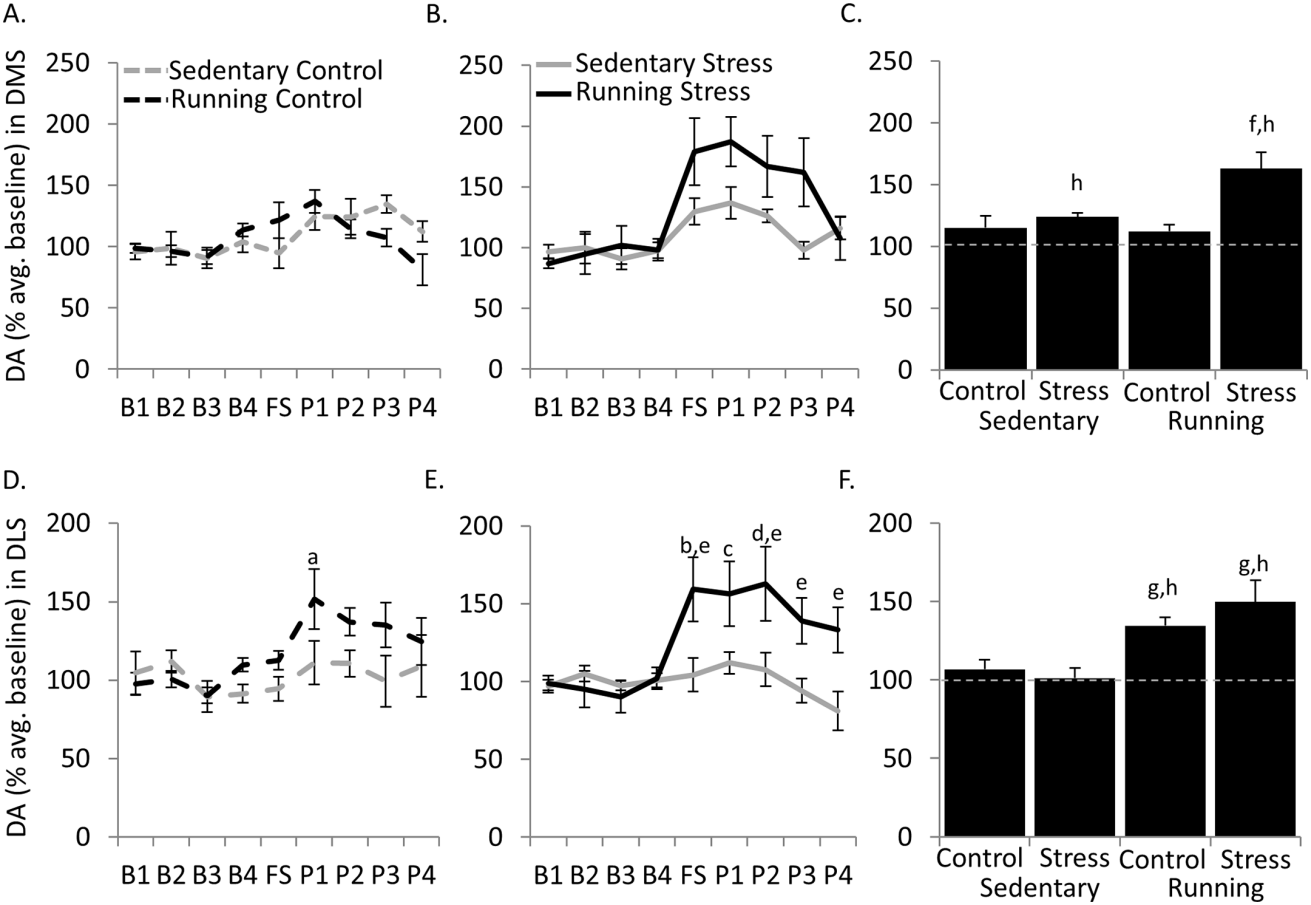
Dopamine concentrations in the dorsal striatum. A) Mean extracellular DA levels expressed as a percentage of baseline samples in the dorsal medial striatum (DMS) of control rats during each 20 min sampling period. B) same as A but for rats exposed to uncontrollable stress (Stress). C) Combined mean post-shock (FS-P4) extracellular DA levels expressed as a percentage of baseline samples (*grey dashed line*) in the DMS for each group. D) Mean extracellular DA levels expressed as a percentage of baseline samples in the dorsal lateral striatum (DLS) of control rats during each 20 min sampling period. E) same as D but for rats exposed to uncontrollable stress (Stress). F) Combined mean post-shock (FS-P4) extracellular DA levels expressed as a percentage of baseline samples (*grey dashed line*) in the DLS for each group. ^a^ *P*<0.05 at P1 from P4 within Running Control; ^b^ *P*<0.05 FS from B2 or B3 within Running Stress; ^c^ *P*<0.05 P1 from B3 within Running Stress; ^D^ *P*<0.05 P2 from B1, B2, or B3 within Running Stress; ^e^ *P*<0.05 at Running Stress from Sedentary Stress for respective sampling period; ^f^ *P*<*0*.*01* from Sedentary Control, Sedentary Stress, Running Control; ^g^ *P*<0.001 main effect of exercise condition; ^h^ *P*<0.01 from baseline (*grey dashed line*)

For individual sampling periods, the 3-way interaction between exercise condition, stress, and sample period was marginally not significant (Fig 3A and 3B [F(25, 206) = 1.54, *P* = 0.06]. However, a main effect of sampling period was found [F(8, 214 = 7.53, *P* < 0.0001]. Moreover, a significant 2-way interaction was found between exercise condition and stress [F(1, 214) = 5.47, *P* = 0.02]. Post hoc analysis on this interaction revealed the Running Stress group maintained overall greater concentrations of DA than Running Control (*P* = 0.001), Sedentary Control (*P* = 0.004), and Sedentary Stress (*P* = 0.01). These group differences are primarily due to the varying degree of increased DA concentrations during the post-foot shock period.

Averaging across the entire post-foot shock sampling periods, a significant 2-way interaction was found between exercise and stress conditions [F(1,26) = 5.46, *P* = 0.03] (Fig 3C). Post hoc analysis revealed that Running Stress group maintained greater post-shock concentrations of DA than Sedentary Control (*P* = 0.009), Sedentary Stress (*P* = 0.03), and Running Control (*P* = 0.005) groups. One group *t-tests* revealed that extracellular DA concentrations tended to be greater during post-foot shock periods than baseline in Sedentary Stress [*t*(7) = 7.06, *P* = 0.0002] and Running Stress [*t*(7) = 4.78,*P* = 0.002] groups (Fig 3C).

#### Dorsal lateral striatum

For individual sampling periods, a significant 3-way interaction between exercise condition, stress, and sample number was found [F(25, 195 = 1.62, *P* = 0.04) (Fig 3D and 3E). Within groups, Running Stress rats had greater DA concentrations at FS compared to B2 (*P* = 0.03) and B3 (*P* = 0.01). Moreover, Running Stress rats had greater DA concentrations at P1 than B3 (*P* = 0.02). Finally, DA concentrations were higher for Running Stress rats at P2 than at B1 (*P* = 0.03), B2 (*P* = 0.02), and B3 (*P* = 0.005). Running Control group had greater DA concentrations at P1 than B4 (*P* = 0.02). Post hoc analysis revealed no differences between groups during B1-B4 sampling periods. However, exercise potentiated dopamine efflux following foot shocks in rats that received tail shock, as Running Stress maintained greater concentrations of DA than Sedentary Stress during FS (*P* = 0.04), P2 (*P* = 0.05), P3 (*P* = 0.02), and P4 (*P* = 0.006) sampling periods.

Averaging across the entire post-foot shock sampling periods, a significant main effect of exercise was observed [F(1, 24) = 15.73, P = 0.0006] (see Fig 3F). No significant main effect of stress or interaction between exercise condition and stress treatment was observed. One group *t-tests* revealed that extracellular DA concentrations tended to be greater during post-foot shock periods than baseline in Running Control [*t*(5) = 6.25, *P* = 0.0015] and Running Stress [*t*(7) = 3.54,*P* = 0.009] groups (Fig 3F).

## Discussion

Results of this study support the conclusion that 6 weeks of wheel running both prevents acute stress induced sensitization of extracellular 5-HT and potentiates extracellular DA in the dorsal striatum following subsequent exposure to foot shocks. Previous work has demonstrated the development of impaired escape behavior 24 h following tail shocks is dependent upon excessive 5-HT activity in the dorsal striatum [30, 49]. Consistent with previous reports, exposure to acute stress potently increased extracellular 5-HT levels in the dorsal striatum of escape deficit vulnerable sedentary rats following exposure to foot shocks [10, 25, 48]. Interestingly, the stress-sensitized 5-HT response was eliminated in wheel running rats, which are also protected from developing escape deficits [10, 23, 48]. Thus, the capacity of wheel running to prevent the sensitized 5-HT response elicited by two foot shocks 24 h following acute stress may provide key insight into the mechanism by which exercise protects against the development of depression-like shuttle box escape deficits.

The exact mechanism by which wheel running prevents acute stress-sensitized 5-HT response following subsequent comparatively mild stress exposure is unknown, but may involve constraining the activation of 5-HT neurons located in the DRN, thereby suppressing the release of 5-HT to projection sites like the dorsal striatum. In support of this hypothesis, our lab has identified a number of targets for plasticity in DRN of wheel running rats that may have influence on the activity of 5-HT neurons, including changes to the expression of 5-HT transporters, 5-HT_1B_R, and 5-HT_1A_R [19]. A potential functional increase of the inhibitory 5-HT_1A_ autoreceptors in the DRN following wheel running is a particularly attractive candidate, since previous work has identified these receptors as a source contributing to the stress-evoked 5-HT response [29, 30, 49]. Moreover, the parallel by which 6 weeks (but not 3 weeks) of wheel access is required to both an increase of 5-HT_1A_R expression in the DRN, and protection against escape deficits that are dependent upon a stress-sensitized 5HT response [19, 48] further suggests increased 5-HT_1A_R from running may contribute to preventing acute stress-evoked 5-HT response [7, 8]. Consistent with this idea, our lab has found 6 weeks of running attenuates acute stress-induced expression of neural activity marker c-Fos in 5-HT neurons in areas of the DRN known to project to the dorsal striatum [10, 48]. However, whether or not functional increases of 5-HT_1A_ autoreceptors contribute to constrained activation of 5-HT neurons in the DRN and subsequent prevention of stress-sensitized 5-HT activity in projection sites, including the dorsal striatum, should be topics for future investigation.

If the mechanism by which wheel running prevents sensitized 5-HT activity in the dorsal striatum is through decreasing the activity of 5-HT neurons across the DRN, it could have broader implications for several behavioral correlates of depression and anxiety related to or dependent upon excessive DRN 5-HT activity including exaggerated fear, reduced social interaction, decreased sucrose preference, and enhanced drug seeking [24, 30, 50, 51]. For instance, the expression of exaggerated fear and reduced social exploration, following exposure to tail shocks, are related to an elevation of extracellular 5-HT in the basolateral amygdala (BLA) and can be prevented by intra-BLA blockade of 5-HT_2C_R or intra-DRN agonism of 5-HT_1A_ autoreceptors [31, 51]. These data indicate acute stress-sensitized activation of 5-HT neurons in the DRN; and excessive 5-HT release in the BLA, a DRN projection site, is responsible for the development of anxiety-like behavior in rats. Therefore, constrained activity of 5-HT neurons in the DRN following wheel running could broadly buffer other stress-evoked depression and anxiety-like behaviors. Whether or not wheel running prevents stress-sensitized extracellular 5-HT concentrations in other DRN projection sites should be topics for further investigation, as it could provide further insight into neural mechanisms of exercise-induced stress robustness.

While the role of acute stress-stimulated 5-HT activity in the development of shuttle box escape deficits is well established, comparatively few studies have explored how excessive 5-HT release may interact with the signaling of other neurotransmitter systems to promote the development of depression-like behavior. Neuroanatomical evidence supports dorsal raphe 5-HT modulation of DA signaling in the dorsal striatum. Indeed, neurons from the DRN are the primary source of 5-HT in the nigrostriatal pathway, which includes DA projections from the substantia nigra to dorsal striatum [52]. DA signaling in the dorsal striatum is critical for the acquisition of goal-directed instrumental processes (as reviewed in [44]), thus altered DA function, as a result of stress-sensitized 5-HT activity, could contribute to shuttle box escape deficits. While some disagreement exists in literature, this hypothesis is generally supported by several lines of evidence indicating augmented DRN 5-HT activity can oppose nigrostriatal DAergic function [36, 37]. Indeed, electrical stimulation of 5-HT neurons in DRN has been reported to inhibit the majority of DA neurons in the substantia nigra [33] and decrease extracellular DA content in the dorsal striatum [34]. In fact, electrical stimulation of dorsal raphe 5-HT neurons at the highest frequency examined (20Hz) decreased extracellular DA levels in the dorsal striatum while lower frequencies (3-10Hz) had no effect [34], suggesting excessive increases in 5-HT levels may be particularly effective at interfering with dorsal striatum DA transmission. Interestingly, evidence suggests activation of 5-HT_2C_R in the dorsal striatum, which are involved in the development of escape deficits following tail shock [25], may also suppress DA release in the nigrostriatal pathway [53], although the circuitry involved is not well established. Therefore, the observed 2–2.5 fold increase of dorsal striatum extracellular 5-HT levels could contribute to a dampening of the DA response in acute stressed sedentary rats following foot shock. Conversely, wheel running prevented stress-sensitized 5-HT activity, which may have eliminated a source of constraint over nigrostriatal DA activity allowing for elevated DA levels following exposure to foot shocks. While it is difficult to conclude that the control of DA transmission following foot shock and during the shuttle box paradigm are similar, given the central role of DA signaling in striatum-involved motor and instrumental learning, the capacity of DA levels to become elevated following subsequent stress exposure may be critical for unhindered escape behavior following stress.

Stress-sensitized 5-HT and -dampened DA levels in sedentary rats, as well as the prevention of these responses by running, were similar in both striatum sub-regions, making it difficult to identify if region specific changes to 5-HT or DA signaling that may underlie the stress-induced development of instrumental learning deficits. However, instrumental learning is now thought to be mediated by two functionally and anatomically distinct modalities, which include goal-directed and habit processes that are under the control of neural substrates comprising the DMS and DLS respectively [38–46]. A growing body of evidence suggests stress exposure mediates instrumental action in a manner that favors the use of well-rehearsed habits over goal-directed strategies (for review see [26]). Consistent with these observations, stress-induced shuttle box escape deficits occur rapidly in rats that have no prior experience with the task (i.e. not rehearsed), suggesting impaired escape behavior may be related to altered signaling in neural circuits responsible for processing goal directed action. Therefore, the possibility remains that stress-sensitized 5-HT or -restricted DA response could be acting in a dorsal striatum region-specific manner to impair shuttle box escape, despite a similar response observed in both the DMS and DLS. Future studies could be aimed at identifying potential dorsal striatum region specific contributions of stress-evoked 5-HT or attenuated DA response to the development of impaired escape behavior.

In conclusion, the current data indicate that 6 weeks of access to running wheels prevents acute stress-induced sensitization of extracellular 5-HT, but potentiates DA concentrations in the dorsal striatum following subsequent mild stress exposure. The ability of physical activity to constrain excessive 5-HT activity from the DRN following stress exposure may allow for the capacity of DA to become elevated in the dorsal striatum. These data may provide key insight into the mechanism by which wheel running protects against shuttle box escape deficits, and may have broader implications for impaired goal-directed processes associated with stress-related disorders like depression.
